# Biotic and Abiotic Soil Properties Influence Survival of *Listeria monocytogenes* in Soil

**DOI:** 10.1371/journal.pone.0075969

**Published:** 2013-10-07

**Authors:** Aude Locatelli, Aymé Spor, Claudy Jolivet, Pascal Piveteau, Alain Hartmann

**Affiliations:** 1 INRA, UMR1347 Agroécologie, Dijon, France; 2 INRA, US-1106 InfoSol, BP20619, Orléans, France; 3 Université de Bourgogne, UMR1347 Agroécologie, Dijon, France; University of Illinois at Chicago College of Medicine, United States of America

## Abstract

*Listeria monocytogenes* is a food-borne pathogen responsible for the potentially fatal disease listeriosis and terrestrial ecosystems have been hypothesized to be its natural reservoir. Therefore, identifying the key edaphic factors that influence its survival in soil is critical. We measured the survival of *L. monocytogenes* in a set of 100 soil samples belonging to the French Soil Quality Monitoring Network. This soil collection is meant to be representative of the pedology and land use of the whole French territory. The population of *L. monocytogenes* in inoculated microcosms was enumerated by plate count after 7, 14 and 84 days of incubation. Analysis of survival profiles showed that *L. monocytogenes* was able to survive up to 84 days in 71% of the soils tested, in the other soils (29%) only a short-term survival (up to 7 to 14 days) was observed. Using variance partitioning techniques, we showed that about 65% of the short-term survival ratio of *L. monocytogenes* in soils was explained by the soil chemical properties, amongst which the basic cation saturation ratio seems to be the main driver. On the other hand, while explaining a lower amount of survival ratio variance (11%), soil texture and especially clay content was the main driver of long-term survival of *L. monocytogenes* in soils. In order to assess the effect of the endogenous soils microbiota on *L. monocytogenes* survival, sterilized versus non-sterilized soils microcosms were compared in a subset of 9 soils. We found that the endogenous soil microbiota could limit *L. monocytogenes* survival especially when soil pH was greater than 7, whereas in acidic soils, survival ratios in sterilized and unsterilized microcosms were not statistically different. These results point out the critical role played by both the endogenous microbiota and the soil physic-chemical properties in determining the survival of *L. monocytogenes* in soils.

## Introduction

*Listeria monocytogenes* is a food-borne pathogen responsible for listeriosis a potentially fatal disease that results in meningitis, septicemia or abortion [1], [2]. This disease can affect humans and a large range of wild and domestic animals [3]. Outbreaks of human listeriosis have been reported worldwide and are mainly associated with consumption of various contaminated food such as meat, dairy products, vegetables and fish [4]–[7]. Ready-to-eat food products, which are consumed without further cooking, are most likely at the origin of listeriosis outbreaks [8]–[11]. Although listeriosis infections are uncommon, mortality rates can reach 30% in at-risk people [12]–[14]. As a consequence, *L. monocytogenes* is recognized as one of the most important food-borne pathogen.

*L. monocytogenes* is widely distributed in nature including vegetation [15], [16], water [17], sediment [18], [19] and soil [15], [20]. Although *L. monocytogenes* is ubiquitous in the environment, human and animals are likely to be an important reservoir [3], [21], [22]. *L. monocytogenes* has been isolated from livestock, domestic and wild animals in both infections and latent states [3], [23], in animal feces and in the close environment of animals [24]. The incidence of *L. monocytogenes* is generally higher in fecal sample of healthy cattle (33%) than in sheep (8%) or pig (5.9%) [3]. This finding is consistent with other studies reporting a significantly higher prevalence of *L. monocytogenes* positive samples in bovine farms than in small ruminant farms without listeriosis cases [24]. In British-fresh livestock manure, prevalence of *Listeria* spp. (including *L. monocytogenes* and *L. ivanovii*) is globally higher in cattle (29.8%) and sheep (29.2%) than in pig (19.8%) and poultry (19.4%) wastes with levels ranging from 2×10^2^ to 1×10^3^ *Listeria* spp. per gram of manure [25].

Farm environments are potential sources of *L. monocytogenes* and may contribute to the contamination of vegetables at the pre-harvest stage. *L. monocytogenes* is frequently isolated from a large variety of vegetables collected in farms [26], [27]. One of the first potential sources of vegetable contamination at the preharvest stage (in the field) is soil when seeds are sown. In addition, some agricultural practices such as recycling animal feces as crop fertilizers or irrigation with contaminated water may increase the risk of soil and vegetable contamination. Soil fertilized with sludge cake can contaminate parsley seeds with *L. monocytogenes* which can be detected until plant harvesting [28]. Finally, direct transfer of *L. monocytogenes* from amended soil to seeds of carrots, lettuce, radish, spinach and tomato has been described [29]. Recent field experiment has shown that the transfer of the pathogen surrogate *Listeria innocua* from contaminated soil to parsley leaves can occur by splashing due to rain and irrigation [30].

Public health hazard linked with transmission of pathogens from soil to plants and vegetables is relevant only if the pathogen is able to survive long enough in soil. Previous studies have investigated which soil properties might impact *L. monocytogenes* survival. Survival studies were performed either by direct inoculation of *L. monocytogenes* in soil or by adding contaminated fertilizer in soil. First, survival of *L. monocytogenes* is not significantly affected by the type of livestock manure added to soil [31], [32]. Soil type had a strong effect on *L. monocytogenes* survival. *L. monocytogenes* survived better in a fertile soil (up to 295 days) than in a clay soil (at 24–26°C) [33]. *L. monocytogenes* population was stable in clay soils, significantly decrease in sandy soils, while displaying an intermediary survival in sandy loam soils up to 30 days [31]. Regardless of the type of manure spread on soil, *L. monocytogenes* persisted over 32 days in a clay loam grassland soil while survival was lower in a sandy arable soil [32]. Soil pH seems to be determinant for *L. monocytogenes* persistence which can survive more than 32 days in 2 soils harboring pH of 6.5 and 6.9 [32]. On the contrary, *L. monocytogenes* EGDe did not persist more than 6 days at 25°C in a forest soil characterized by a low pH (5.22) [34]. The rapid decline observed in this study, can be explained by the low pH of the soil. Higher survival of *L. monocytogenes* was observed at low temperature [34]. Soil microflora appears to have an impact on *L. monocytogenes* survival. Generally, suppression of microflora via soil sterilization allowed a better growth of *L. monocytogenes* than in the presence of a competitive microflora [31], [34], [35]. Biotic and abiotic soil parameters also affect the persistence in soils of pathogenic bacteria belonging to the Enterobacteriaceae family, for example, *Escherichia coli* and *Salmonella enterica* [32], [36], [37].

Generally, studies reporting the survival of pathogenic bacteria in soil, including *L. monocytogenes*, were focused on a limited number of poorly characterized soils [31], [32], [35]–[37]. Identification of the soil abiotic and biotic parameters influencing the survival of pathogenic bacteria in terrestrial ecosystems will help understanding their cycle of contamination in the environment. The objective of this study was to assess the survival of *L. monocytogenes* in a large collection (n = 100) of well characterized soils collected throughout France and representative of the pedology and land use of the whole territory. ANOVA and variance partitioning were used to correlate 40 soil parameters with *L. monocytogenes* survival ratios in order to identify parameters that determine the fate of *L. monocytogenes* in soil.

## Materials and Methods

### Soil Samples

One hundred soil samples were randomly chosen among the soil library of the French Soil Quality Monitoring Network (RMQS, Réseau de Mesure de la Qualité des Sols) [38], [39]. Soils were collected from 2001 to 2010 following a single sampling procedure. Soil sample preparation and storage were achieved according to ISO and AFNOR standards : NF ISO 10381-1 [40] and ISO 10381-6 [41]. A large range of physical (particle-size) and chemical parameters (pH, organic C, N, exchangeable cations and cation exchange capacity (CEC)…) were measured for each soil by the Soil Analysis Laboratory of INRA (Arras, France, http://www.lille.inra.fr/las). In addition, 2 variables were calculated from measured parameters: (i) C/N ratio and (ii) Base Cation Saturation Ratio (%) determined by the sum of Ca^2+^, Mg^2+^, Na^+^, K^+^ divided by CEC and multiplied by 100. Supplementary information such as climatic data (monthly rain, evapotranspiration (ETP) and temperature) and detailed land cover are available at the DONESOL database (http://www.gissol.fr/programme/rmqs/RMQS_manuel_31032006.pdf [42], [43]). All parameters characterizing the 100 soils used in this study, including soil texture, soil chemistry, land-use, climatic data and spatial localization are detailed as supporting information (Table S1). Moreover, the textural classification of the 100 soils is represented in supplementary figure (Figure S1).

### Bacterial Strain and Culture Conditions

The rifampicin-resistant (Rif^R^) mutant L9 of wild-type strain *L. monocytogenes* EGD-e was used in this study [44]. Stock cultures were prepared by growing *L. monocytogenes* Rif^R^ in Tryptone Soy Broth (TSB, AES Chemunex, Bruz, France) at 37°C. After washing in sterile water, the cell pellet was suspended in a Brain Heart Infusion (BHI, AES Chemunex, Bruz, France) with 25% of glycerol, aliquoted (200 µl in microtubes) and frozen −80°C until further utilization. Pre-cultures were prepared by inoculating 20 ml of TSB inoculated with 200 µl of the stock culture. After 48H of incubation at 20°C, pre-cultures were centrifuged (10,000 g, 5 min), pellets were washed and re-suspended in 20 ml of physiological saline solution (8.5 g/L NaCl). Cell density was estimated spectrophotometrically (a cell suspension with an OD_600nm_ of 1 was considered to contain 1.9×10^9^ *L. monocytogenes* cells per ml), and pre-cultures were diluted with physiological saline solution to a final inoculum concentration of 1.6×10^7^ *L. monocytogenes* per ml. The exact concentration of the final inoculum was determined by plate count on TSB medium.

### Microcosms Preparation and Inoculation

Microcosms were prepared in sterile flasks (40 ml) using 2 grams of carefully mixed and homogenized soil taken from the initial stock (100 grams).

To test the effect of biotic factors, a subset of nine soils were sterilized by gamma-radiation. Two grams of soil were conditioned in a 40 ml-flask. For each soil, 9 individual microcosms were prepared (3 sampling days and 3 replicates). All 81 (9×9) soil microcosms were packed in a box and send to Ionisos for gamma radiation sterilization (Dagneux, France). The entire box was sterilized without being open by receiving an external minimum dose of 45 KGray and an external maximum dose of 60 KGray.

Soil microcosms were adjusted to 80% of the water field capacity one week before inoculation. Water field capacity was determined by granulometric method taking into account clay, fine silt and organic matter content of each soil. For all tested soils, one individual microcosm was prepared for each sampling time. One hundred and twenty µl of inoculum was added to each microcosm giving concentrations of 1×10^6^ *L. monocytogenes* per gram of dry soil. Due to the large number of soils tested only one repeat was realized, however the reproducibility of the method was evaluated on a subset of 9 soils for which three repeats were realized. On the subset of nine soils, three independent flasks were inoculated for each soil condition (sterilized or non-sterilized). For each sterilized soil, uninoculated microcosms remained sterile for the whole duration of the experiment (84 days), thus proving that gamma irradiation eradicated all microorganisms and that no recontamination of microcosms occurred during the experiment. Soil microcosms were incubated at 20°C and were sampled 1 h (*i.e.* survival ratio at t_0_), 7, 14 and 84 days after inoculation. All viable counts were expressed per gram of dry soil.

Total cultivable soil bacteria were enumerated on one tenth-strength Nutrient Agar medium supplemented with 100 mg/L of cycloheximide for non-sterile soil microcosms at each sampling time (from day 0 to day 84 after inoculation).

### Monitoring *L. monocytogenes* Survival by Viable Plate Count

Eighteen ml of Tryptone Salt (TS, 1 g/L tryptone, 8.5 g/L NaCl) was added to each microcosm and bacteria were resuspended by shaking 10 min, 150 rpm, 20°C. This suspension was serially diluted and plated on Polymyxin-Acriﬂavin-Lithium-Chloride-Ceftazidime-Aesculin-Mannitol agar (PALCAM, AES Chemunex, Bruz, France) supplemented with rifampicin and cycloheximide each at 100 mg/L and incubated at 37°C. Appropriate dilutions were plated on PALCAM medium. When high levels of *L. monocytogenes* are expected in soil, 10×10 µL were spotted for each dilution level (Detection limit = 100 bacteria per gram of dry soil), while when less than 100 *L. monocytogenes* per gram of soil was expected, 1 ml of the soil suspension was poured into the medium (Detection limit = 10 bacteria per gram of dry soil). Uninoculated control experiments were processed accordingly to verify that *L. monocytogenes* was not initially detected in uninoculated soils.

### Statistical Analyses of the Survival Data

Survival ratios were calculated as follows prior to the analyses to make them closely conform to a Gaussian distribution:
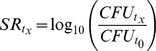
Where 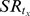 is the survival ratio at time *t_x_* (*x* = 7 days, 14 days or 84 days), 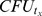 is the number of Colony Forming Units at time *t_x_* and 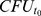 is the number of Colony Forming Units at time *t_0_*.

### Replicated Experiment : pH, Land Use and Sampling Day Accounting for Variation of *L. monocytogenes* Survival Ratio in Sterilized and Non-sterilized Soils

A repeated-measures ANOVA was performed on the survival ratios. Factors accounting for variation of the survival ratio were the pH of the soils (treated as an ordinal variable: pH <5.5; 5.5<pH <7; pH >7), the presence or absence of soil microflora (non-sterilized versus sterilized soil), the land use (culture, forest and grassland) and the sampling time, the latter being the repeated factor. Pair-wise differences between levels of factors were assessed using a *t*-test. Bonferroni corrections were used to take into account multiple comparisons.

### Ecological Survey: Partitioning of the Biological Variation for Survival Ratio in the 100 Soils

Forty different soil properties and environmental parameters were used in this analysis (Table S1). To identify the soil edaphic and the environmental factors driving *L. monocytogenes* survival in soils, all the explaining variables measured were first grouped into five categories: soil texture, soil chemistry, land use, climate and spatial coordinates.

Spatial vectors were constructed using the Principal Coordinates of a Neighbor Matrix approach (PCNM, [45]). This method was applied to the geographic coordinates of the different sampled sites and yielded 59 spatial vectors. All quantitative explanatory variables were transformed using Box-Cox transformation prior to analyses (the corresponding lambda parameters were estimated by maximum likelihood [46]).

For each 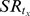, significant explanatory variables, as well as PCNM vectors, were chosen by model selection (forward and backward) and by minimizing the Akaike Information Criterion (AIC). Statistical significance was assessed by 1999 permutations of the reduced model. The respective contribution of contextual variables (explanatory variables or combinations of explanatory variables) was assessed using redundancy analysis ordination [47]. All these analyses were performed with R using the vegan package (functions *pcnm*, *varpart* and *rda*).

## Results

### Identification of Soil Abiotic (Physico-chemical) Properties Impacting *L. monocytogenes* Survival in a Panel of 100 Soils

Total cultivable bacteria were enumerated for each soil at day 0, day 7, day 14 and day 84. At day 0, total number of bacteria range from 4.78×10^6^ to 1.07×10^8^ CFU per gram of soil. We found that the total bacterial community counts remain stable (within the same order of magnitude) over the 2-month experiment period for all investigated soils (Student’s t test, p = 0.265).

As soils were stored for varying periods, a regression analysis between *L. monocytogenes* survival rates and soil sampling date (year) was done. This analysis revealed no correlation between these two variables (data not shown). The age of soil samples doesn’t not seem to influence *L. monocytogenes* survival.

Survival of *L. monocytogenes* was determined in a collection of 100 soils. *L. monocytogenes* population globally declined with time in the 100 soils. In 71% of the soils, *L. monocytogenes* was detected until the end of the experiment (i.e. 84 days after inoculation) and final populations ranged from 10 to 1.27×10^4^ CFU per gram of soil. In 21% of the soils, survival was observed only at short term (up to day 7 or up to day 14). Finally, in 8% of the soils, *L. monocytogenes* was not detected 7 days after inoculation.

To identify soil edaphic and environmental factors driving *L. monocytogenes* survival in soils, partial regression models were calculated for the 5 categories of explaining variables (Table 1). Using this approach, we were able to explain from 46.6% to 79.5% of the survival observations. Most of the variance of the survival ratios was explained by 3 groups of variables, *i.e.* soil chemistry, soil texture and spatial localization. Climate and land use do not appear in the model (Table 1) because they do not explain any variance of the survival ratios of *L. monocytogenes*. Soil chemistry was relevant to explain short-term survival ratios at days 7 and 14 (64.5% and 65.4%) of *L. monocytogenes* in soils (Table 1). When studying the effect of each variable independently, Basic Cation Saturation Ratio (BCSR) was identified as the major soil chemical characteristic determining short-term survival profiles (day 7 and day 14, Table 2) and differences in soil BCSR explained up to 55.4% of the variability of *L. monocytogenes* survival. Cationic exchange capacity (10.3%) in one hand and exchangeable Ca (11%) in the other hand further explained survival ratio of *L. monocytogenes* at day 7 and day 14, respectively. Soil texture was the contextual variable with the highest weight for explaining long-term survival profiles (up to day 84), as it explained 11% of the observed variance (Table 1). When studying the effect of each variable independently, clay content explaining 30.9% of the observed variance seems to be the major contributor followed by exchangeable Al (Table 2). Interestingly, pH was not found to be a significant contributing factor to *L. monocytogenes* survival but this is explained by the fact that BCSR and pH are strongly but non-linearly correlated as shown in supporting figure (Figure S2). Hence, variation in survival ratio captured by the BCSR cannot be attributed to the pH in the partial regression analysis. In other terms, if the BCSR was removed from the analysis, pH would be selected as a significant explaining variable.

**Table 1:** Partitioning of the variation of survival ratios of *Listeria monocytogenes* as a function of contextual variables.

		Overall model			% explained variance of the contextual variables		
		N^a^	F-ratio	Explained variance^b^ (%)	Soil Chemistry	Soil Texture	Spatial Distance
Survival at	Day 7	13	30.64***	79.5	64.5***	2**	0.5°
	Day 14	5	43.02***	67.1	65.4***	–	1.6°
	Day 84	8	27.64***	46.6	1.3^NS^	11.1***	4.2*

a N is the number of explanatory variables retained after selecting the most parsimonious explanatory variables (by minimizing the Akaike Information Criterion).

b the % explained variance corresponds to the adjusted R^2^ values of the overall model using partial redundancy analysis.

NS Non Significant, °p<0.1, *p<0.05, **p<0.01 and ***p<0.001.

Note that the covariation between the contextual variables is not reported in this table, therefore summing over the different contextual variables does not give the % explained variance of the overall model.

- Soil texture do not explain any variance of the survival ratio of *L. monocytogenes* at day 14.

**Table 2:** Contribution of the five most important explanatory variables to the variation in survival ratios of *Listeria monocytogenes*.

		% Variance explained by:				
*Survival at*	*Day 7*	BCSR (55.4%)	CEC (10.3%)	Coarse Silt (4.6%)	Sp. Dist*_V6_* (2.6%)	Al*_exch_* (1.8%)
	*Day 14*	BCSR (47.7%)	Ca*_exch_* (11%)	Mn*_exch_* (4.9%)	Al*_exch_* (4.6%)	Sp. Dist*_V13_* (1.1%)
	*Day 84*	Clay (30.9%)	Al*_exch_* (5.5%)	CaCO_3tot_ (3.5%)	Sp.Dist*_V3_* (2.9%)	Temp_month_ (2.7%)

The respective contributions of each variable were calculated by taking into account all the significant variables in the model using partial redundancy analyses (significance assessed with 1999 data permutations).

BCSR : Basic Cation Saturation Ratio, CEC : Cation-Exchange Capacity, Sp. Dist*_V_* : Spatial Distance correspond to the spatial vector *x* from the PCNM analysis, Al*_exch_* : exchangeable aluminum, Ca*_exch_* : exchangeable calcium, Mn*_exch_* : exchangeable manganese, CaCO_3tot_ : Total calcareous content,Temp_month_ : mean temperature per month.

### Evaluating the Impact of the Interactions between Biotic and Abiotic Soil Parameters on *L. monocytogenes* Survival

**Figure 1:**
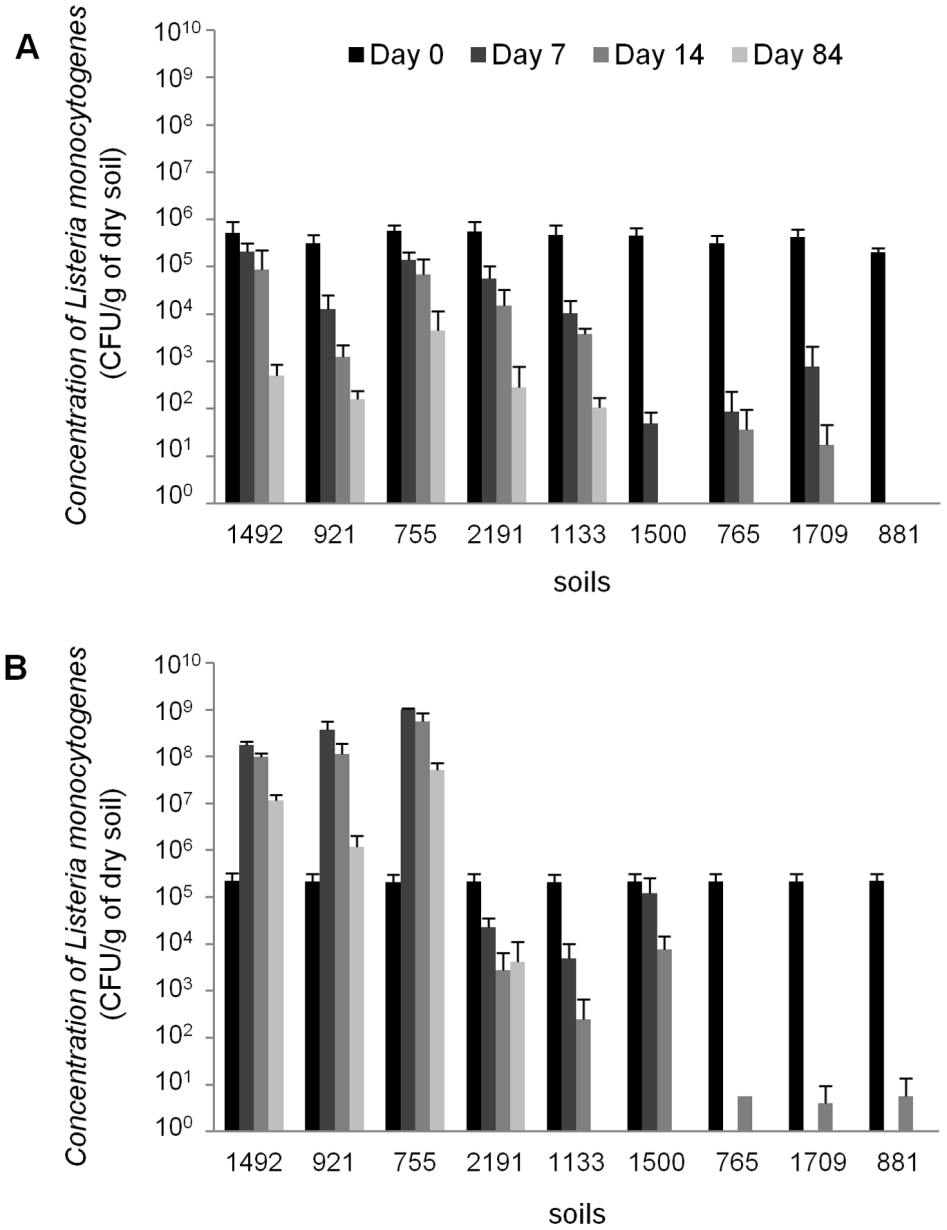
Survival of *Listeria monocytogenes* in nine soils: non-sterile (panel A) and sterile (panel B). Black, dark grey, medium grey and light grey bars represent *L. monocytogenes* population density (CFU per gram of dry soil) at Days 0, 7, 14 and 84 after inoculation, respectively. Error Bars indicate the mean ± standard deviation over three replicated measurements.

Focusing on a subset of 9 soils chosen for their contrasted physico-chemical properties and land use characteristics (Table 3), we evaluated the impact of the soil endogenous microbiota on the survival ratio of *L. monocytogenes* in a replicated complete block design. Statistical analysis by ANOVA allowed first to validate the reliability of inoculation and counting method and the repeatability between experiments. *L. monocytogenes* population globally declined with time in non-sterile soils. However, significant differences in the survival profiles were observed between the nine different soils (Figure 1, panel A). Long-term survival was observed after inoculation in 5 soils (n° 1492, 921, 755, 2191 and 1133) and the population of *L. monocytogenes* range from about 10^2^ to 4×10^3^ bacteria per gram of soil at the end of the experiment (84 days). In three other soils (soils n°1500, 765, and 1709) survival did not exceed 7 to 14 days after inoculation, while in soil n° 881, *L. monocytogenes* was no longer detected as soon as 7 days after inoculation. Results showed that the survival of *L. monocytogenes* was different between sterile soils (Figure 1, panel B). Indeed, 1 to 3 log growth was observed in soil n° 1492, 921 and 755. For the others soils, no growth of *L. monocytogenes* was observed and *L. monocytogenes* abundance decreased. In these soils, both short and long-term survival profiles were observed as already noticed with-non sterile soils.

**Table 3:** Land use and main edaphic factors of the subset of nine soils.

Soil n°	Land use	pH	Clay content (g/kg soil)	Silt content (g/kg soil)	Sand content (g/kg soil)
**765**	Grassland	4.7	121	164	715
**1709**	Grassland	4.7	185	288	527
**2191**	Grassland	5.9	374	440	186
**921**	Culture	7	454	508	38
**1492**	Culture	7.9	403	223	374
**1500**	Culture	5.3	150	210	640
**755**	Forest	7	650	334	16
**881**	Fore**s**t	4.7	153	446	401
**1133**	Fore**s**t	5.6	819	92	89

**Figure 2:**
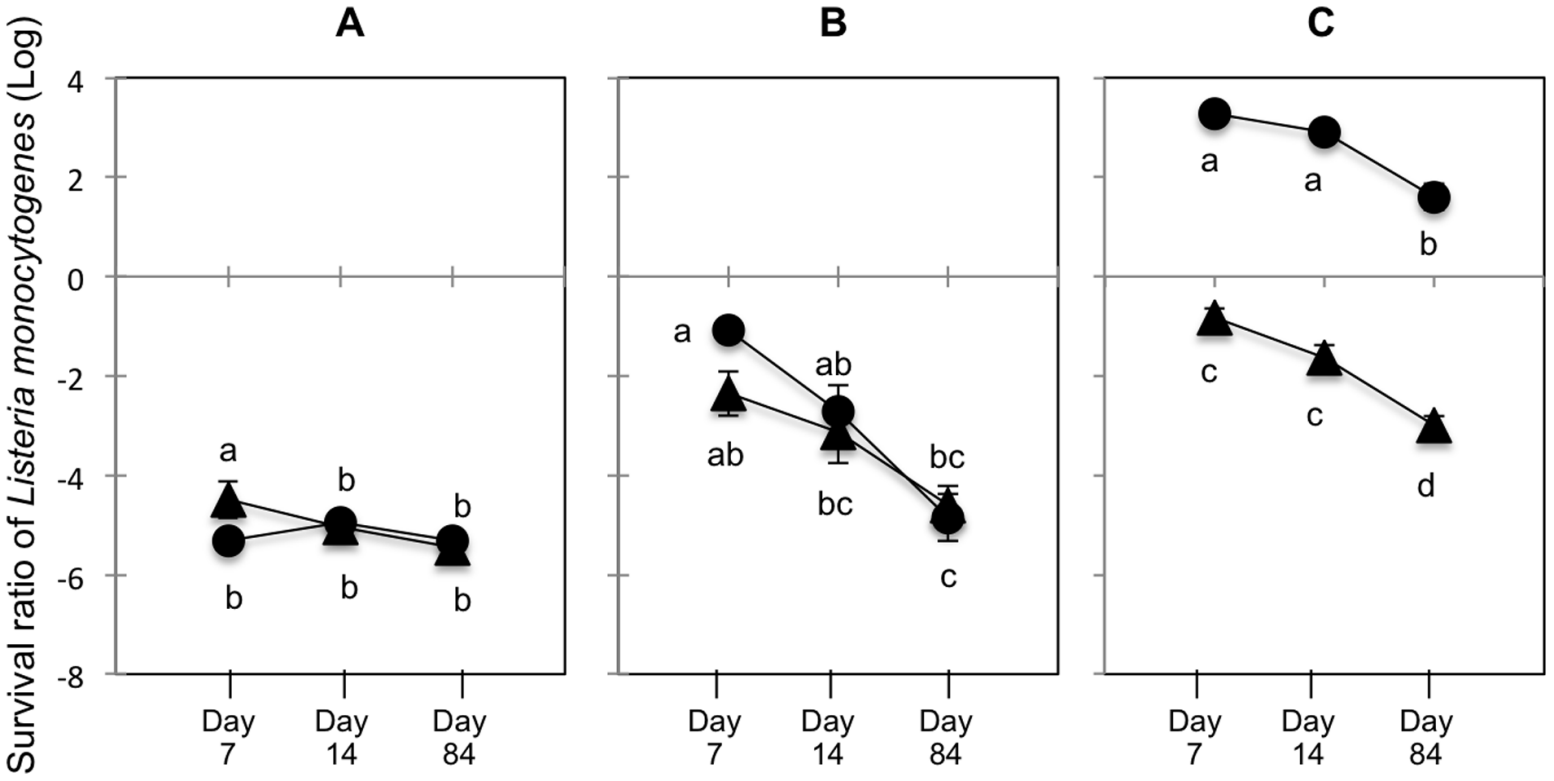
Mean survival ratios of *L. monocytogenes* in non-sterile and sterile soils depends on soil pH. The nine soils were distributed in three equal classes function of their pH with (A) 3 soils with pH <5.5, (B) 3 soils with 5.5<pH <7 and (C) 3 soils with pH >7. Mean survival ratios were calculated between soils belonging to the same pH classes for each sampling time. Triangles represents mean survival ratio in non-sterile soils and circles represented mean survival ratio in sterile soils. Error bars represent the mean ± standard deviation of triplicate experiments.

Using repeated-measures ANOVA, we investigated whether pH or land use could be factors interacting with the soil microbiological status and accounting for the different survival profiles observed over the experiment time course (Table 4). We found a strong and significant interaction between pH and the microbiological status of the soil (*F_2,132_* = 55.03, *p*<0.001) that explains a large part of the observed variance in *L. monocytogenes* survival ratio. The 3-way interaction integrating time is also significant (*F_4,132_* = 4.47, *p* = 0.002) indicating that differences in survival ratio between pH classes for the sterilized and the unsterilized soils vary over time. Globally, *L. monocytogenes* survival is higher in the highest pH class of soils, than in lower pH classes of soils. Moreover, the suppressive role of the endogenous microbiota on its survival is clearly evidenced in the high pH class and growth of *L. monocytogenes* populations was observed in sterilized soils grouped in the highest pH class (Figure 2).

**Table 4:** Analysis of variance for *Listeria monocytogenes* survival ratios in a subset of nine soils.

	Survival Ratio		
*Sources of Variation*	*df*	*Sum of Squares*	*F*
*pH*	2	608.15	471.88***
*Microbiological status* a	1	99.20	153.94***
*Land use*	2	21.16	16.42***
*Day of sampling*	2	90.77	70.44***
Land use*Microbiological status	2	13.31	10.33***
Land use*Day of sampling	4	1.85	0.72
pH*Microbiological status	2	70.92	55.03***
pH*Day of sampling	4	25.77	9.99***
Microbiological status*Day of sampling	2	0.27	0.21
Land use*Microbiological status*Day of sampling	4	10.68	4.14**
pH*Microbiological status*Day of sampling	4	11.53	4.47**

df: degrees of freedom, F: Fisher’s F, .

a correspond to sterilized versus non-sterilized soils.

*** p<0.001,

** p<0.01.

We also found a significant 3-way interaction between land use, microbiological status of the soil and time of sampling (*F_4,132_* = 4.14, *p* = 0.003) on *L. monocytogenes* survival. The highest survival was observed in soils collected from culture fields while it is lowest in grassland (sterilized or unsterilized). However, land use classes overlapped to some extent with pH classes. For example, two out of the three culture soils belong to the high pH class and two out of the three grassland soils belong to the low pH class, the forest soils being the only well-spread out in the different pH classes. For these reasons, pH appeared to be more likely (than land use) an explaining factor determining *L. monocytogenes* survival in soils. This study based on a small subset of soils allowed us to identify the pH and the endogenous microbial communities as important factors influencing *L. monocytogenes* survival in soils.

## Discussion

Circulation of pathogens in the farm environment may generate health hazards [26], [27], [48]. Regarding food-borne pathogens, pre-harvest contamination may be a source of food contamination, thus increasing the risk of outbreaks. The presence of the food-borne pathogen *L. monocytogenes* in soil has been connected with pre-harvest food contamination [29], [30], [49] and its survival in soil has been documented [31]–[34] but limited information is available on the soils used in these studies and edaphic factors that may affect its survival are poorly understood. Previous studies focused on a limited number of soils including from 1 to 3 soils [32], [33]. Moreover, in these studies, soils were mainly characterized by their textural classes and only a few chemical characteristics were available and were used in statistical analysis [31], [32]. In this study, we investigated the survival of *L. monocytogenes* in a large set of 100 well-characterized soils (chemical and textural characteristics) representative of the French territory and occurring worldwide. Indeed, the variation of the age of the soil samples is inherent to a study based on a very large set of soils. In our study, the age of soil samples did not explain any variation of the survival rates of *L. monocytogenes*. This result suggests that soil storage did not modify significantly soil composition and thus did not introduce a major bias in the experiment. Moreover, there is no significant quantitative variation of total soil bacterial populations over the 2 month’s experiment for all soil tested.

We observed a decline of *L. monocytogenes* populations with time in all 100 soils that is consistent with *L. monocytogenes* behavior reported in previous studies [31], [33]–[35]. Moreover, in the present study, we demonstrated that the survival ratio depended on the soil under scrutiny and overall three trends were evidenced: long-term, short-term and lack of survival. Indeed, in most soils (71%), *L. monocytogenes* was still detected at the end of the experiment (84 days); this long-term survival is in agreement with previous studies relating *L. monocytogenes* survival until 200 days in a clay soil and 295 days in a “fertile” (as stated by authors) soil [31], [33]–[35]. In 21% of soils, the pathogen was no longer detected after two weeks. Finally, in 8% of soils, a dramatic decline to undetectable levels occurred within the first week of incubation. A similar observation was reported in a forest soil by McLaughlin *et al*. [34]. Identification of edaphic factors that may explain these trends is difficult as biotic and abiotic parameters are intertwined, however our study demonstrated clearly and for the first time that *L. monocytogenes* is able to survive in a majority of soils.

We used intentionally large numbers of bacteria for the inoculation of soil samples to facilitate short term monitoring of declining populations. This might introduce a bias since the survival of larger bacterial population requires higher quantities of nutrients. However, Dowe *et al*. (1997), using two levels of inoculum (10^2^ and 10^6^ bacteria per gram of soil) have demonstrated that after a short period, *L. monocytogenes* counts reached similar levels with both inocula [31]. This result indicates that *L. monocytogenes* survival in soil is not durably affected by the size of the inoculum.

The statistical model we developed could explain most of the variance of *L. monocytogenes* short-term survival (67% at day 14 and 80% at day 7) suggesting that the comprehensive characterization of our soil samples is adequate to explain short-term survival ratio. The soil chemistry was the most significant contextual variable and, among these variables, the Base Cation Saturation Ratio (BCSR) was the most impacting on short-term survival. BCSR was calculated as a ratio between the sum of exchangeable base cations (Ca^2+^, Mg^2+^, K^+^ and Na^+^) and CEC. The CEC of a soil is the total quantity of exchangeable cations that a soil can fix and release at a specific pH.

Contrarily to short-term survival, the model was less efficient in explaining variations of long-term survival (47% at day 84) this suggests that the soil abiotic characteristics that fed the model were not sufficient to explain long-term survival. We can hypothesize that soil microflora is one of the variables missing and that biotic factors are critical for long term survival. However, soil texture and more precisely clay content appear to be the principal variables explaining long-term survival variance among the abiotic factors analyzed. (Figure S1).

Until now, only a few studies have shown that soil texture had an impact on *L. monocytogenes* survival [31]–[33]. The survival of other pathogens such as *E. coli* or *Salmonella* spp. was also shown to depend on the soil texture [32], [36], [37], [50]. In general, these studies demonstrated that finer-textured (clayey) soils result in prolonged survival of introduced bacterial pathogens compared to coarser-textured (sandy) soil.

The experiment conducted on a subset of 9 soils (comparison of sterile versus non sterile soil) evidenced the major role of soil microflora in controlling *L. monocytogenes* survival. Our results are in agreement and reinforce previous studies [31], [34], [35] showing that suppression of the microflora allowed the growth of *L. monocytogenes* in soils. Unexpectedly, no *L. monocytogenes* growth was observed in sterilized microcosms for 6 out of the 9 soils tested in our study. This result strongly suggests that abiotic characteristics of these soils were non-permissive for the growth (and survival) of *L. monocytogenes*. The repeated-measures ANOVA pointed out to pH as the major factor explaining this lack of growth. Indeed, we found a strong and significant interaction between pH and soil microflora on *L. monocytogenes* survival. More precisely, the suppressive effect of soil microflora on *L. monocytogenes* survival was the strongest in soils with high pHs. Soil pH has already been identified as a structuring and even predictive parameter for the composition and structure of global bacterial communities’ [51], [52]. Bacterial richness and diversity appears to be higher in neutral soils and lower in acidic soils [51], [53]. Similarly, pH seems to be critical for the fate of *L. monocytogenes* in soil, since the survival ratio is higher in neutral soils.

In our study, we demonstrate that chemical properties of soils (in particular BCSR) explain most of the variability of short-term survival (soil texture explaining mostly long-term survival). However, it is established that soil chemistry is tightly linked to soil texture. The BCSR reflects the amount of base cations present in soil as well as the number of negative sites supplied by the soil matrix. Clay particles and organic matter are both constituents of soil that harbor negative charges, which can fix and release positively charged nutrients including cations. So, clay and organic matter content largely determine the CEC and the BCSR of a soil. Most of the studies investigating the effect of soil texture on bacterial pathogens survival highlight the fact that finer-textured soils with high clay content are more favorable to bacterial pathogens survival than coarser-textured soils. This trend can be explained by a higher availability of pore spaces protecting bacteria from protozoan predation. Our result further complete this explanation proving that the finer soil texture with high clay content will also help to maintain a sufficient base cations pool essential for bacterial life. This hypothesis can be confirmed by the result of the long-term survival, which is correlated with clay content.

Only plate counts were used to monitor *L. monocytogenes* survival. This might lead to underestimate *L. monocytogenes* concentration if Viable But Not Culturable (VBNC) cells of *L. monocytogenes* are present [54], [55]. Indeed, VBNC state can be induced for *L. monocytogenes* in response to low pH and nutrient limitation [55]–[57]. These conditions (low pH and starvation) may occur in some soil microcosms. Occurrence of VBNC *L. monocytogenes* may induce inconsistencies of the model to explain variations of *L. monocytogenes* survival (especially for survival at 84 days). Counting VBNC bacteria in non sterile soil samples is extremely difficult, however, this might be achieved in the future.

Overall, this is the first extensive study of the survival of *L. monocytogenes* in a large collection of well-characterized soils. We found that the pool of cations that soil can exchange is an indicator of *L. monocytogenes* short-term survival, that population decline is faster in acidic soils and finally that the presence of the microflora participates to the barrier effect of soil towards invasion by *Listeria monocytogenes*. Further work should aim at deciphering which members of the soil microflora are critical to explain *L. monocytogenes* survival. This might be realized on a limited subset of soils chosen for contrasted characteristics.

## Supporting Information

Figure S1 **Distribution of the 100 soils in the textural triangle.** Each dot corresponds to one soil. Survival ratio of *L. monocytogenes* in soils at day 84 are expressed as the grey level of each dot (light grey corresponding to low survival ratios and dark grey corresponding to high survival ratios).
(TIF)

Figure S2 **Relationship between pH and corresponding BCSR of the 100 non-sterile soils.** Each dot corresponds to one of the 100 soils tested.
(TIF)

Table S1 **Soil parameters, including soil texture, soil chemistry, land-use, and climatic data characterizing the 100 soils used in this study.**
(XLSX)
