# Supplementary figures and images for: Biotic and Abiotic Soil Properties Influence Survival of Listeria monocytogenes in Soil
Source: PLoS One. 2013 Oct 7;8(10):e75969. doi: 10.1371/journal.pone.0075969 (PMC3792134; doi:10.1371/journal.pone.0075969)

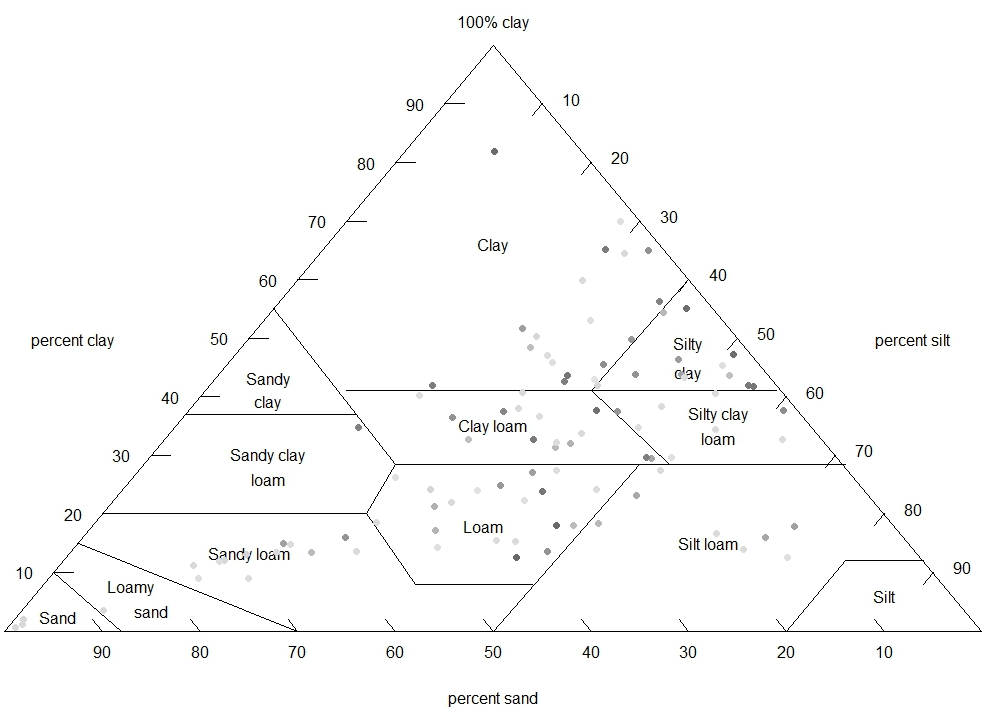

Supplement: Figure S1 — Distribution of the 100 soils in the textural triangle. Each dot corresponds to one soil. Survival ratio of L. monocytogenes in soils at day 84 are expressed as the grey level of each dot (light grey corresponding to low survival ratios and dark grey corresponding to high survival ratios). (TIF) [file pone.0075969.s001.tif]
